# Staphylococcal Superantigen (TSST-1) Mutant Analysis Reveals that T Cell Activation Is Required for Biological Effects in the Rabbit Including the Cytokine Storm 

**DOI:** 10.3390/toxins2092272

**Published:** 2010-09-09

**Authors:** Norbert Stich, Martina Waclavicek, Nina Model, Martha M. Eibl

**Affiliations:** 1 Biomedizinische ForschungsgmbH Lazarettgasse 19/2, A-1090 Vienna, Austria; Email: Norbert.Stich@biomed-research.at (N.S.); Martina.Waclavicek@biomed-research.at (M.W.); Nina.Model@biomed-research.at (N.M.); 2 Immunology Outpatient Clinic, Schwarzspanierstrasse 15, A-1090 Vienna, Austria

**Keywords:** sepsis, inflammation, T cell involvement, superantigens, endotoxin

## Abstract

Staphylococcal superantigens (sAgs), such as toxic shock syndrome toxin 1 (TSST-1), induce massive cytokine production, which may result in toxic shock syndrome (TSS) and sepsis. Recently, we reported that *in vitro* studies in human peripheral blood mononuclear cells (PBMC) do not reflect the immunological situation of the host, because after exposure to superantigens (sAgs) *in vivo*, mononuclear cells (MNC) leave the circulation and migrate to organs, e.g., the spleen, liver and lung. Our experimental model of choice is the rabbit because it is comparable to humans in its sensitivity to sAg. T cell activation has been assessed by lymphocyte proliferation and IL-2 gene expression after *in vivo* challenge with TSST-1 and the mutant antigens; expression of the genes of proinflammatory cytokines were taken as indicators for the inflammatory reaction after the combined treatment with TSST-1 and LPS. The question as to whether the biological activities of TSST-1, e.g., lymphocyte extravasation, toxicity and increased sensitivity to LPS, are mediated by T cell activation or activation by MHC II-only, are unresolved and results are contradictory. We have addressed this question by studying these reactions *in vivo*, with two TSST-1 mutants: one mutated at the MHC binding site (G31R) with reduced MHC binding with residual activity still present, and the other at the T cell binding site (H135A) with no residual function detectable. Here, we report that the mutant G31R induced all the biological effects of the wild type sAg, while the mutant with non-functional TCR binding did not retain any of the toxic effects, proving the pivotal role of T cells in this system.

## 1. Introduction

Infections caused by *Staphylococcus aureus* (*S. aureus*) represent a health problem of increasing importance. Due to the growing prevalence of antibiotic resistance to these bacteria, new treatment options are urgently required to combat the disease. The severity of *S. aureus* sepsis is positively associated with staphylococcal toxins with properties of a superantigen (sAg) [1], e.g., TSST-1. In contrast to conventional antigens, sAg bind to major histocompatibility complex (MHC) II molecules outside the peptide-binding cleft and to the T cell receptor (TCR) variable β chains (Vβ) on the T cell. The hypothesis has been put forward that cross-linking MHC II and the TCR induces inflammation characterized by a cytokine storm [2]. 

A cytokine storm followed by strong inflammation can cause widespread tissue damage and multiorgan failure [3,4]. The relatively weak *in vivo* and *in vitro* responses to sAg of mice compared with humans have been attributed to differences between mouse and human MHC II, thereby emphasizing the important role of MHC II [5,6].

Several attempts have been undertaken to detoxify TSST-1 by mutating either their TCR binding site (e.g., mutation of histidine 135 to alanine) [7] where the MHC binding site remains functional or the MHC II binding site (e.g., mutation of glycin 31 to arginine) with functional binding to TCR [8]. Results obtained with these mutants or from binding studies were contradictory in part. Some reports showed that inhibition of the interaction sAg-TCR could stop excessive inflammation [9,10], whereas others underlined the importance of MHC II binding [11]. Whether sAg-MHC II binding or TCR-sAg interaction is of decisive importance for activation of innate immunity has to be further clarified.

Schlievert *et al.* showed that streptococcal mutant sAgs lacking residues necessary for T cell activation still retained lethality in rabbit models [12]. These authors tested a series of single and double mutants showing that the ability of exotoxins to cause lethality and endotoxin enhancement does not require superantigenicity.

Dinges *et al.* showed that impairment of T cell function by cyclosporin protected rabbits against the lethality of toxic shock syndrome (TSS) [13]. In contrast, they showed that total body irradiation could not prevent TSS. However, they could not exclude the possibility that the results might have been skewed by a small number of radio-resistant T cells.

Inflammation by LPS is strongly enhanced after priming with sAg [14]. The mechanism responsible for this sensitization is not well understood. It has been suggested that the upregulation of TLR4 by sAg might be responsible for the enhanced response to LPS [15] as TLR4 is the receptor for LPS and this mechanism of innate immunity might be responsible for the overwhelming inflammatory response that leads to tissue damage [16].

Kum *et al*. emphasized the importance of sAg/TCR interaction based on *in vitro* studies. They studied the temporal sequence of the cytokine expression pattern in human peripheral blood mononuclear cells (PBMC) after stimulation with wild type (wt) TSST-1, G31R and H135A. Using enzyme-linked immunoabsorbent assays (ELISAs), they showed that G31R induced a cytokine profile similar to that of wtTSST-1, while H135A showed complete absence of any cytokine secretion [9]. According to our knowledge, the biological effect of these mutants has never been studied *in vivo*. 

SAgs are known to strongly increase sensitivity to LPS. Rabbits pretreated with even low doses of sAg die when exposed to low amounts of LPS [13]. The mechanism of this amplification is still poorly understood. 

In our studies, using the mutant toxins G31R and H135A *in vivo,* we explored whether, in addition to MHC class II binding, T cell binding was mandatory for the sensitization for LPS. 

Recently, applying quantitative real time PCR (qRT-PCR) and proliferation assays, we detected cell activation and strong induction of cytokine gene expression in the organs of rabbits soon after sAg administration. This activation could not be detected in the periphery [17]. Therefore, results obtained from the circulation do not accurately reflect the immunological state of the host. 

Here we studied those biological effects of staphylococcal toxins (superantigens) of which the mechanisms of action are not well understood. We applied the mutants G31R and H135A and prove that T cell activation is mandatory for both Vβ unrestricted extravasation and for the sensitization for LPS. 

## 2. Results and Discussion

### 2.1. G31R induces leukopenia, lymphopenia and monocytopenia in rabbits. In contrast, no effect on MNC subsets by H135A

Rabbits were studied in terms of their leukocyte and lymphocyte counts after administration of the TSST-1 mutants G31R and H135A. To compare the induced effects with the ones elicited by the wt protein, we used TSST-1 as a positive control. Negative control rabbits were only injected with PBS.

**Figure 1:**
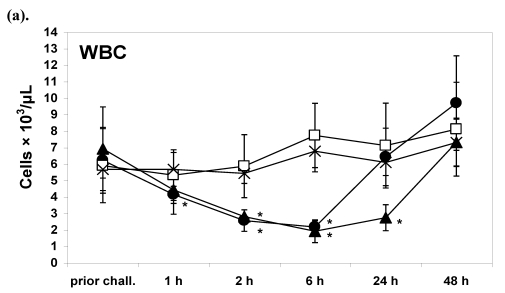

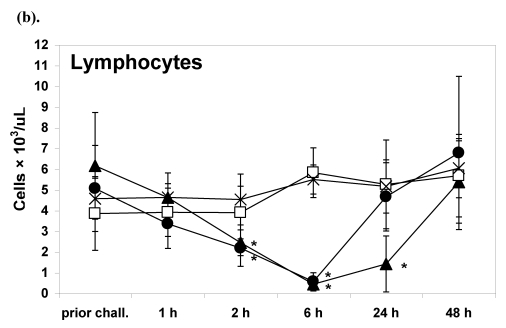

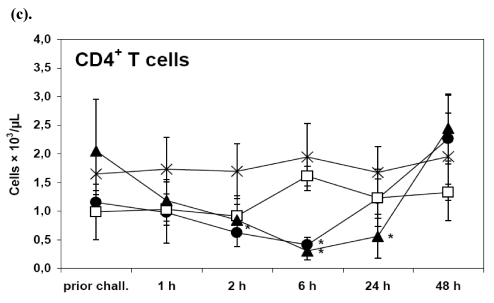

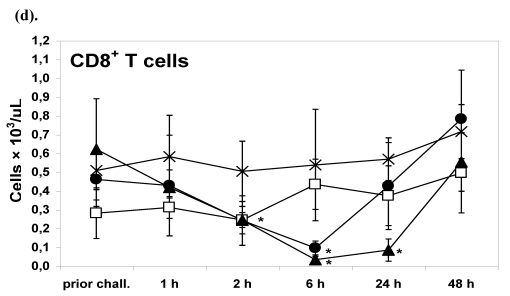

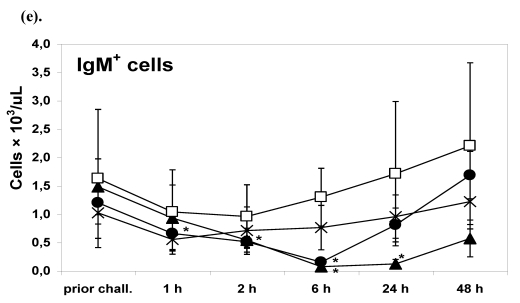

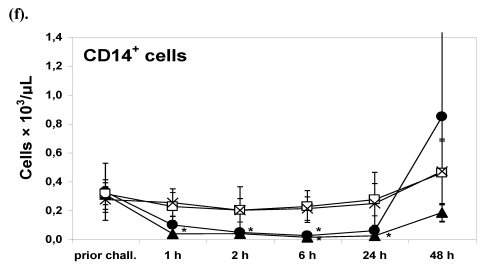
(**a**–**f**) Significant decrease of CD4^+^, CD8^+^, IgM^+^ and CD14^+^ cells in the circulation of rabbits injected with G31R or TSST-1. No influence on cell counts of WBC and subsets after application of H135A. Rabbits were injectedwith either 100 µg G31R (n = 6) [●] or 100 µg H135A (n = 6) [□]. The positive controls received TSST-1 (n = 4) [▲], PBS (n = 4) [X] and the negative controls received PBS (n = 4) [X]. Prior to application, as well as 1 h, 2 h, 6 h, 24 h and 48 h after injection, the numbers of WBC (**a**) and total lymphocytes (**b**) were determined from venous blood of rabbits. The absolute numbers of the lymphocyte subsets (**c**) CD4^+^, (**d**) CD8^+^, (**e**) IgM^+^ and (**f**) CD14^+^ were calculated from FACS analyses at the time points denoted, as described in Materials and Methods. Figures (**c**–**f**) show mean values ± SD of the numbers of the indicated cell populations of 4 rabbits injected with G31R, and of 4 rabbits administered by H135A. Mean ± SD of positive and negative control rabbits were calculated from 4 experiments.

Prior to injection, WBC of rabbits (n = 20) tested were 6150 ± 1905 cells/µL (AV ± SD). After application of G31R, the number of circulating leukocytes started to decline and decreased to 2171 ± 378 cells/µL (AV ± SD) 6 h after injection (Figure 1A). Thus, the counts of WBC were comparable to the levels reached 6 h after TSST-1 application (1913 ± 688 cells/µL). The leukocytes within the circulation returned to baseline levels already 24 h after G31R injection which was slightly earlier than after TSST-1 application. No significant changes were observed in H135A-treated rabbits or in the controls (Figure 1A). Similar results were obtained with total lymphocyte counts after administration of G31R, TSST-1 *versus* H135A and PBS (Figure 1B).

Flow cytometric studies of different lymphocyte subsets revealed that the effects at G31R on the number of CD4^+^ and CD8^+^ T cells as well as CD14^+^ and IgM^+^ cells within the circulation were comparable to the effects induced by TSST-1 (Figure 1C-1F). However, after G31R injection, cell counts of the studied subsets increased earlier as compared to TSST-1 suggesting a quantitative difference. 

These results indicate extravasation of monocytes and lymphocytes by G31R comparable to TSST-1, while no effect was observed after injection of H135A under identical conditions (Figure 1A-1F).

### 2.2. T cell activation by proliferation assays in human PBMCs can be detected after stimulation with wtTSST-1 and G31R but not with H135A

**Figure 2:**
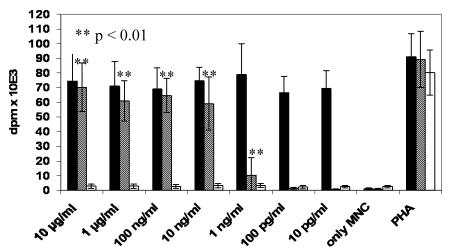
T cell activation and proliferation of human PBMC is induced by G31R and TSST-1. No proliferation of human PBMC after stimulation with H135A. Human PBMC derived from healthy donors were cultured for 5 days in the presence of the indicated concentrations of TSST-1 (n = 4, black bars), G31R (n = 6, hatched bars) and H135A (n = 4, open bars). Columns represent mean values ± SD of dpm as a measure of 3H-thymidine uptake of cells. As a positive control, PBMC were stimulated by PHA.

The mitogenic effects of TSST-1 and several mutants of TSST-1 have been studied in several species. Furthermore, TSST-1 and the mutants G31R and H135A have been compared in terms of their T cell activation by monitoring the percentage of Vβ2^+^ CD25^+^ expressing cells in flow cytometry studies [27]. Here, we compare the dose response of T cell activation by measuring the proliferative capacity of human PBMC. We found that G31R significantly stimulated the proliferation of T cells in a range from 10 µg/mL–1 ng/mL as compared to medium. This was lower than proliferation induced by TSST-1, whereas H135A was devoid of mitogenic capacity in the whole range (Figure 2).

### 2.3. IL-2 expression is induced in the organs after administration of wtTSST-1 and G31R but not after administration of H135A

**Figure 3:**
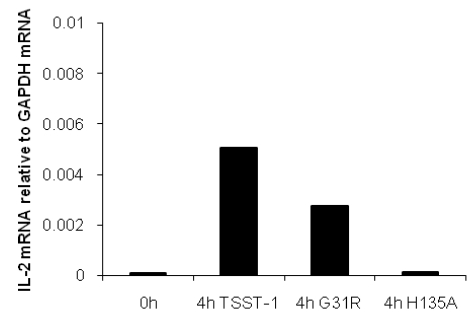
IL-2 gene activation in the spleen after administration of TSST-1 and G31R. Rabbits were challenged with G31R, wtTSST-1, and H135A for 4h and the spleen was extracted as described in Materials and Methods. IL-2 expression was determined via real time PCR and was related to the expression of the house-keeping gene GAPDH by dividing the IL-2 mRNA copy numbers through the mRNA copy numbers of GAPDH.

Injection of G31R resulted in an induction of IL-2 gene expression in the spleen (Figure 3). The induction of IL-2 gene expression was similar after application of wtTSST-1. By contrast, no induction of IL-2 gene expression was noted in the spleen when H135A was given. 

### 2.4. TSST-1 and G31R induce inflammatory cytokines in the organs

As previously shown [17], cytokine gene expression is significantly induced in the organs after application of TSST-1. Here we addressed the question whether the mutants of TSST-1 induced different cytokine profile compared to that of the wildtype.

**Figure 4:**
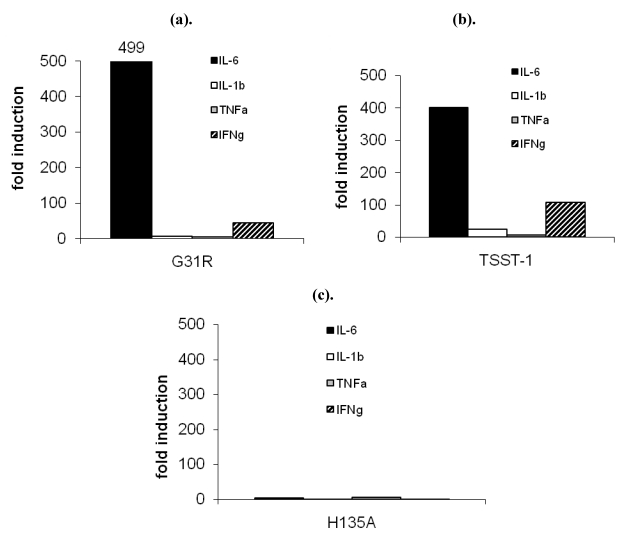
(**a**–**c**) The expression of proinflammatory cytokines is induced in the spleens of rabbits after a challenge with G31R and wt TSST-1. Rabbits were challenged for 4 h and the expression of the proinflammatory cytokines was measured in the spleen by real time PCR. (**a**) G31R and (**b**) TSST-1 strongly increase the expression of IL-6 (black bars, ■) and IFNγ (hatched bars, 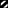). Induction of IL-1β (white bars, □) and TNFα (grey bars, 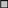) was weak (6-fold and 5-fold). (**c**) H135A did not cause an induction of cytokines.

Figure 4A shows the cytokine expression in the spleen after administration of G31R. We detected a cytokine expression pattern similar to that observed after application of TSST-1. IL-6 (black bars) was strongly induced (500-fold) and also IFNγ gene expression (hatched bars) was significantly enhanced (50-fold). IL-1β (white bars) and TNFα (grey bars) were slightly increased (6-fold and 5-fold, respectively).

The induction levels after administration of TSST-1 were comparable (Figure 4B). IL-6 (black bar) was most strongly induced (400-fold) but also IFNγ (hatched bar) was significantly induced (100-fold). Inductions of IL-1β (white bar; 24-fold) and TNFα (grey bar; 7-fold) were less. Induction of gene expression was also observed in other organs (liver, lung) but it was considerably less than in the spleen (data not shown).

By contrast, application of H135A did not significantly increase any cytokine expression (Figure 4c).

### 2.5. G31R- and H135A- pre-priming differ in the induction of the cytokine storm after LPS triggering

**Figure 5:**
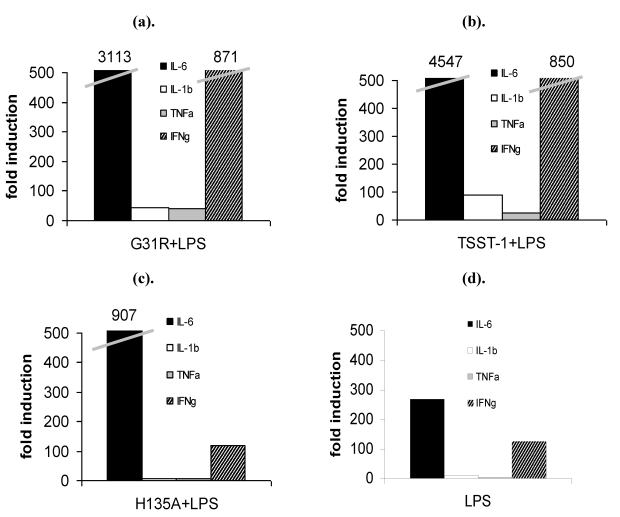
(**a**–**d**) Pre-priming with TSST-1 leads to a strong enhancement of LPS sensitivity and to a subsequent cytokine storm in the spleen. Rabbits were challenged for 4 h with wtTSST-1, G31R and H135A followed by administration of LPS for a further 2 h. The expression of proinflammatory cytokines was quantified with real time PCR. (**a**) G31R + LPS and (**b**) TSST-1 + LPS induce an exorbitant expression of pro-inflammatory cytokines, particularly of IL-6 (black bars, ■) and IFNγ (hatched bars, 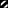). IL-1β (white bars, □) and TNFα (grey bars, 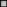) were less induced. There was no induction of cytokines in rabbits treated with (**c**) H135A + LPS in comparison to (**d**) LPS-challenged animals.

LPS administration after TSST-1 priming leads to a cytokine storm in the organs. Here we show that G31R + LPS (Figure 5A) led to a cytokine storm. IL-6 was induced 3113-fold, IFNγ 871-fold, IL-1β and TNFα both 50-fold. These levels were comparable to those observed after TSST-1 + LPS treatment (Figure 5B). By contrast, H135A + LPS (Figure 5C) did not give rise to significant cytokine gene induction. The induction was slightly stronger than after application of LPS alone (Figure 5D). Table 1 summarizes the fold induction levels of the proinflammatory cytokines in the spleen.

### 2.6. Discussion

There is general agreement that animal models available at present are sub-optimal for understanding the sequence of events in sepsis. Even though it is generally agreed upon that infection by one or several microbial agents is responsible for pathogenesis, the diagnosis of sepsis still greatly relies on clinical symptoms of inflammation and, in spite of greatly improved methodology in microbiology, the identification of an underlying infection, proven by the detection of bacteremia, is still lacking in 30–50% of cases. 

Even though the amplification of endotoxins (LPS) of Gram-negative bacteria by exotoxins of Gram-positive bacteria (with characteristics of sAg) is clearly established, this notion has not been taken into consideration in animal models of sepsis. Extensive colonization of the population with Gram-positive bacteria and the relevance of the interrelation between colonizing and invasive infection in septic patients is well recognized [18]. 

Investigation in infants who died of SIDS (Sudden Infant Death Syndrome) revealed that these infants were significantly more often, more extensively double colonized with *S. aureus* [19] and *E. coli* [20]. We have raised the hypothesis that a similar mechanism may be operational in sepsis because of the frequent colonization with toxin-producing *S. aureus* and endotoxin-producing and releasing *E. coli* in the gut. Evidence of considerable contact with toxin-producing staph is provided by the fact that over 90% of adults have antibodies to at least one, but usually several, staph toxins. TSST-1 potentiates the activity of endogenous endotoxin by inhibiting clearance from the blood [21]. We applied the combined treatment of the staph exotoxins, e.g., TSST-1, and endotoxin of Gram‑negative bacteria, e.g., LPS of *E. coli*, as our experimental model in order to delineate the cellular sequence of the events in this system. 

The rabbit was selected as the species most appropriate for these studies because of its high sensitivity to staph sAg. Although most animal studies have been performed in mice, sensitivity of mice to these sAg is extremely low. Application of 200 µg/kg of TSST-1 followed by 400 µg/kg of LPS was needed to cause a fatality rate of 50% in mice, whereas 10 ng/kg TSST-1 and 10 µg/kg LPS were required to bring about a fatality rate of 100% in rabbits [22]. Notably, lethality developed at approximately the same peak circulating level of TNFa in mice and rabbits showing that both species are equally sensitive to the lethal effects of circulating TNFa.

Furthermore, exposure to the toxin-producing bacteria and/or the toxin itself causes a disease in rabbits similar to human TSS. Therefore, as previously pointed out [17,23], the rabbit model has been preferred to answer questions regarding the pathophysiology of TSS. 

In the rabbit model we started to analyze the interrelation between T cell activation and inflammation: assessing T cell activation by lymphocyte proliferation and IL-2 gene expression and inflammation by activation of the genes of proinflammatory cytokines (IL-6, TNFα, IL-1β and IFNγ). 

In a previous paper, analyzing the effect of TSST-1 *in vivo* in rabbits, we described that application of sAg leads to Vβ-unrestricted migration of MNC from the circulation [17]. Activated cells have been detected in organs, e.g., the spleen, the liver and the lung. The compartmentalization of the response after exposure to sAg is important but often neglected. Cytokines produced (early) in the organs, such as TNFα in the spleen [3,24], might not be detected in the periphery. Other authors described that lymphocytes leave the circulation in a Vβ-dependent manner [25]. Thus, mechanisms of this extravasation are not well understood in this context. As the migrating cells (activated T cells, B cells, and cells of the monocyte macrophage lineage) all express MHC II on their surface, it was unclear whether direct interaction between sAg and these cells via MHC II would be sufficient to trigger the process. Leukopenia and lymphopenia were described after sAg challenge in different species and lymphopenia is also a frequent finding in patients with TSS and septic shock caused by *S. aureus*. Results of experiments with the mutant toxins clearly indicate that sAg-TCR interaction is a mandatory step for extravasation. The G31R mutant with the intact TCR binding site induced extravasation of lymphocytes comparable to TSST-1 (Figure 1). In contrast, the mutant H135A with intact MHC II binding, but defect in TCR interaction, had no effect (Figure 1). Our analysis of the mutants G31R and H135A confirmed the results of Kum *et al*. [9] describing that the mutant G31R induced significant T cell activation, albeit of lower magnitude than TSST-1 in human MNC. 

**Table 1:** Fold induction levels of proinflammatory cytokines in the spleen after administration of sAg, LPS or the combination.

Cytokine	Toxin	Fold induction
IL-6	TSST-1	401
IL-6	G31R	499
IL-6	H135A	3
IL-6	TSST-1 + LPS	4547
IL-6	G31R + LPS	3113
IL-6	H135A + LPS	907
IL-6	LPS	268
IFNγ	TSST-1	107
IFNγ	G31R	44
IFNγ	H135A	0
IFNγ	TSST-1 + LPS	850
IFNγ	G31R + LPS	871
IFNγ	H135A + LPS	120
IFNγ	LPS	124
IL-1β	TSST-1	24
IL-1β	G31R	6
IL-1β	H135A	0
IL-1β	TSST-1 + LPS	90
IL-1β	G31R + LPS	44
IL-1β	H135A + LPS	5
IL-1β	LPS	11
TNFα	TSST-1	7
TNFα	G31R	5
TNFα	H135A	1
TNFα	TSST-1 + LPS	26
TNFα	G31R + LPS	39
TNFα	H135A + LPS	7
TNFα	LPS	4

Cells activated by sAg migrate from the circulation to the organs, e.g., the spleen and the liver. IL-2 gene expression could be detected early after TSST-1 challenge mainly in the spleen of rabbits [17]. Similarly, T cells from the spleen were described to be the source of early TNFα in response to SEB in D-galactosamine-sensitized mice [3]. 

Here, we report induction of IL-2 gene expression in the spleen of rabbits treated with G31R and wtTSST-1 while no increased expression could be detected with H135A. These findings led us to ask the question whether T cell activation, *i.e.*, IL-2 gene expression, is correlated with the amplification of the inflammatory response induced by LPS. To the best of our knowledge, there are no *in vivo* reports available describing that rabbits pre-treated with TSST-1 reacted with a cytokine storm in the organs when LPS was given later. Induction of proinflammatory cytokine genes, mainly IL-6 but also IFNγ, IL-1β and TNFα, was higher than observed after treatment with either toxin alone. These proinflammatory cytokines are mainly produced by non-T cells, mainly macrophages and dendritic cells. The results clearly indicate that the primary signal is given by T cells leading to activation of IL-6 expression by macrophages and dendritic cells. It has been shown in mouse [15] *in vivo* and in human cells *in vitro* [26] that sAgs enhance the TLR4 expression on monocytes. We also observed a slight increase of TLR4 gene expression in rabbits and mice following administration of TSST-1 (unpublished results). This enhanced expression of TLR4 may be responsible for the augmented susceptibility to its ligand LPS. 

Moreover, sAg may also modulate the interaction between staph and other entities of innate immunity, e.g., TLR2, which may be of importance in clearing staph infections.

The mutant H135A did not induce T cell proliferation or IL-2 gene expression. Thus, this mutant did not have the potential for T cell activation. This mutant did not induce extravasation of lymphocytes and monocytes, nor did it sensitize for the LPS-triggered cytokine burst, proving that T-cell activation is mandatory for these biological effects. Even though the combined effect of sAg and LPS has rarely been studied, and not in the same system, these results are in agreement with the report of Kum *et al*. [9] and differ from the studies of Roggiani *et al.* [16]. The reason for this difference could be explained by the different sAg applied (streptococcal *versus* staphylococcal) and by the different experimental setup. Other studies drew their conclusions from *in vitro* experiments [9] or from studies performed in mice [27] or rats [28].

## 3. Experimental Section

### 3.1. Animals

New Zealand white female rabbits 1.5–2 kg were purchased from Charles River Laboratories (Sulzfeld, Germany). Animals were kept in standard care facilities according to the guidelines of the Austrian Ministry for Science and Research and had free access to food (Altromin 2120 standard diet pellets; Marek Futtermittelwerke, Vienna, Austria) and water. The animal experiments had been approved and controlled by the Veterinary Department of the City of Vienna. 

### 3.2. Injection of the toxins

100 µg of the wt TSST-1, G31R, H135A and 10µg of LPS were applied for time periods as indicated in the figures. The substances were dissolved in 1 mL of PBS, sterile filtered and injected as previously described [17]. For control experiments, PBS was either subcutaneously (2 × 0.5 mL) or intravenously (1 × 1 mL) administered.

### 3.3. Organ extraction, RNA isolation and reverse transcription

Organ extraction after anaesthesia of rabbits was performed at time points indicated in the figures and was extensively described in a previous report [17]. Organs were then homogenized using an Ultra-Turrax T8 homogenizator (IKA, Staufen, Germany) and RNA was extracted according to the protocol of the manufacturer. 900 ng of RNA was reversely transcribed using the 2xRT Kit from Invitrogen (Invitrogen, Paisley, UK). 

### 3.4. Substances

TSST-1, G31R and H135A were produced in our laboratory. Both the expression and purification of TSST-1, G31R and H135A are extensively described by Gampfer et al. [29]. All substances were tested for their biological activities in our laboratory and were proven to be endotoxin-free.

### 3.5. Antibodies for flowcytometry analyses

The murine unlabeled or conjugated mAbs recognizing the rabbit cell surface antigens CD4 (clone: KEN-4), CD8 (clone: 12C7), IgM (clone: NRBM) and CD14 (clone: Tsk4) were purchased from Serotec (Oxford, UK). The corresponding IgG1 and IgG2a negative control mAbs were obtained from BD Pharmingen (Becton Dickinson, San Jose, CA, USA).

### 3.6. Immunofluorescence analyses

Venous EDTA-whole blood samples drawn before and 1 h, 2 h, 6 h, 24 h and 48 h after injection were analyzed for their leukocyte and lymphocyte counts with the help of a Coulter counter machine (Beckman Coulter) and a hematology analyzer (Abbott Laboratories, Abbott park, IL, USA) followed by flow cytometry. Immunofluorescence analyses were performed as previously described [17]. In short, 100 µL whole blood was incubated with mAbs specific for CD4, CD8, CD14, IgM or irrelevant isotype-matched mAbs. After incubation, cells were washed and binding of the primary mAb was visualized using rabbit F(ab')2 anti-mouse Ig-fluorescein isothiocyanate (FITC) (STAR9B; Serotec). Subsequently, cells were lysed, washed and analyzed on a FACS Calibur flow cytometer supported by the CELLQUEST software (Becton Dickinson). FACS-data allowed determining the percentage of granulocytes and mononuclear cells (MNC) within these whole blood samples by gating cells according to their size and granularity. Consequently, absolute granulocyte numbers were calculated. The knowledge of the calculated absolute MNC numbers, and the percentage of CD4^+^, CD8^+^, IgM^+^ and CD14^+^ cells within the MNC population, allowed determination of absolute cell numbers of these subpopulations within the whole blood samples.

### 3.7. Lymphocyte proliferation assay

Peripheral blood mononuclear cells (PBMC) were isolated from heparinized blood of healthy human adults using density gradient centrifugation with LymphoprepTM (Axis-Shield PoC, Oslo, Norway), as previously described [17]. In brief, cells (1 × 10^5^ cells/well) were cultured in 96-well flat‑bottom tissue culture plates (Sarstedt, Newton, NC, USA) in complete medium consisting of RPMI 1640 medium (PAA laboratories, Pasching, Austria), 10% FCS (HyClone, Logan, UT, USA), 2 mM L-glutamine (Invitrogen, Paisley, UK), 100 U/mL penicillin, and 100 µg/mL streptomycin (Invitrogen). PBMC were stimulated in triplicate by rTSST-1, G31R and H135A used in final concentrations between 10 µg/mL and 10 pg/mL. As a positive control, phytohaemagglutinin (PHA, Sigma-Aldrich, St. Louis, MO, USA) were used in the final dilution of 1:320. Stimulated cells were cultured for five days in a humidified atmosphere (37 °C, 5% CO_2_). On day four, 0.5 µCi/well 3H‑thymidine (GE Healthcare, Chalfont St Giles, UK) was added and 18 h later cells were frozen and stored at −20 °C until harvesting onto glass fiber filters. Incorporated radioactivity was determined on a MicroBeta Trilux 1450 scintillation counter (Wallac, Turku, Finland) and expressed as dpm.

### 3.8. Primers

Gene-specific oligonucleotide primers were designed by hand and by primer design Primer Express® v2.0 software from Applied Biosystems (Foster City, CA, USA). Where genomic sequence information of rabbit was not available, consensus sequences of the cytokine genes from human and mouse served for primer design. Primer pairs were synthesized at MWG Biotech (Heidelberg, Germany) and at Invitrogen.

### 3.9. Preparation of external cDNA standards

CDNA standards were prepared as previously described [17] and served for absolute quantification of the target samples.

### 3.10. QRT-PCR and quantification

Quantitative Real time PCR has been extensively described [17]. In short, cDNA from samples and standards were simultaneously amplified on the same plate using an ABI Prism 7700 (Applied Biosystems, Foster City, CA, USA) using the SYBR Green Super Mix from Invitrogen with a ROX reference dye. At the end of the amplification, a melting curve analysis was performed.

The number of target cDNA copy numbers in the cellular samples was then calculated by creating a standard curve where the cycles at threshold (CT) were plotted against the logarithmic values of the cDNA standard copy number. GAPDH served as an internal standard. Fold induction of gene expression was assessed from values normalized for the expression of GAPDH and then related to the mean values derived from the spleen of three unchallenged rabbits.

### 3.11. Statistical analyses

Paired t-test has been applied to calculate the significance of the decrease between pre- and post‑application values of WBC, lymphocytes and lymphocyte subsets. A decrease was considered to be significant at a p value ≤ 0.05 and is marked with an asterix.

Students’ t-test has been applied to evaluate the significance (p ≤ 0.01) in proliferation experiments.

## 4. Conclusions

In conclusion, we prove *in vivo* that following exposure to sAg, T cell activation is the mandatory first step for the inflammatory/toxic effects studied. The most pronounced cause of toxicity is the cytokine storm in sepsis and it has been suggested that the cytokine storm is mainly responsible for toxicity. The majority of cells involved in the production of proinflammatory cytokines are non‑T cells, such as B cells and cells of the monocyte/macrophage lineage. Our findings demonstrate the primary role of T cells in activation and/or triggering of these non-T cell populations. T cells have been shown to modulate the inflammatory reaction. The understanding of the mechanism of T cell modulation of the inflammatory response in this context is of pivotal importance and is presently under investigation in our lab.
